# “It didn't feel like they cared”: exploring factors that influence privacy and disclosure in disordered eating and eating disorders (DEEDs)

**DOI:** 10.1186/s40337-025-01467-9

**Published:** 2025-12-27

**Authors:** Ashleigh N. Shields, Nichole Alejandro, Calabria DeFazio, Caitlin Laska, Jennifer L. Evans

**Affiliations:** 1 Department of Public Health and Health Sciences, Northeastern University, 360 Huntington Avenue, Boston, MA 02115 USA; 2 Department of Communication Studies, Northeastern University, 43 Leon St #212a, Boston, MA 02115 USA; 3 University of Connecticut, 233 Glenbrook Rd, Unit 4100, Storrs, CT 06269 USA

**Keywords:** Eating disorders, Disordered eating, Privacy management, Disclosure

## Abstract

**Background:**

Disordered eating (DE) affects millions of individuals each day as they are exposed to diet culture, normalization of restricting foods, and public perception of a "healthy" body and diet. Persistent DE behaviors may progress in severity and frequency, leading to harmful behaviors that result in physical and psychological health outcomes, ultimately meeting the diagnostic criteria for an eating disorder (ED). The purpose of this study was to identify what factors influence disordered eating and eating disorders (DEED), why individuals do not disclose their experiences, and what could have been done to help them with their DEED.

**Methods:**

Using the Communication Privacy Management theory to develop questions, qualitative semi-structured interviews were conducted with 15 participants. A thematic analysis was conducted from interview transcripts to develop overarching themes.

**Results:**

Five key themes emerged from participant interviews: family influence and comments, social media influence, healthcare influence, justification factors, and comorbidities. These themes reflect influences on participants developing DEEDs and barriers to getting help such as stigma associated with mental health issues and healthcare providers' unwillingness to discuss their DEED. Many participants also noted that family influences, particularly mothers, were a contributing factor to their DEED as well as playing sports and social media like Tumblr or "What I eat in a day" videos on TikTok.

**Conclusion:**

These findings highlight the complex social, cultural, and structural factors that shape privacy management and the development and disclosure of DEED. Public health professionals can use these results to help bridge the gap between education, policy reform, and accessible healthcare to address the often-overlooked public health issues of DEED.

## Abstract (plain language summary)

Disordered eating and eating disorders (DEEDs) impact many individuals and when left unattended can lead to deadly consequences. In this study, we asked people about what influenced or continues to influence their DEED behaviors, why people did not tell those close to them about their DEEDs, and what they wish people close to them would have done to help them. What we found is that there are a lot of influences on DEEDs mostly stemming from childhood and adolescence. The people we talked to in this study said their family was a big influence, especially their mothers who played a large part in their development of DEEDs as well as their grandparents and fathers. We also learned that social media has played a role by seeing people make DEEDs look glamorous or through "What I eat in a day" videos. Some individuals said that healthcare providers also influenced their DEEDs by not taking their patients seriously about DEED concerns. Often participants mentioned their DEED was coupled with mental health challenges, that are often stigmatized. These influences also dissuaded participants from telling those close to them about their struggles, which prevented them from seeking help. This research helps us understand what leads to DEEDs and what prevents people from getting the help they need.

## Introduction

Within the United States, 30 million individuals [1] will experience an eating disorder (ED), which is defined as "serious illnesses marked by severe disturbances in a person's eating behaviors" [2], p.1. Eating disorders and similar behaviors like disordered eating (DE) remain pervasive in public health, affecting individuals across the lifespan and contributing to significant psychological and physical health consequences. This disorder is perpetuated through exposure to messaging that emphasizes the normalization of restrictive eating, diet culture, and perceptions of what a "healthy" body looks like. This may cause individuals to engage in DE behaviors, which may include dieting, skipping meals or fasting, restricting foods and food intake; using diuretics, weight loss medications, or laxatives; and engaging in purging or excessive exercising [3–5]. When DE behaviors persist and become severe and more frequent, they can affect relationships, daily life, and have serious psychological and physiological health issues, ultimately meeting the diagnostic definition for an ED [6]. Rates of Disordered Eating and Eating Disorders (DEEDs) are rising, with a global ED prevalence increasing from 3.5% to 7.8% between 2000 and 2018 [2]. EDs can have deadly consequences for anyone regardless of their age, race, ethnicity, or sex, and have considerable costs to the individual, their family, and overall society [7]. Developing DEEDs depends on a variety of risk factors, which include psychological, biological, and social factors. *Psychological* factors include perfectionism, anxiety, depression, body dissatisfaction, history of substance abuse, impulsivity, and obsessive thoughts [8–12]. *Biological* factors involve having a close relative with past ED experience and/or mental health conditions, and a history of dieting [1, 13, 14]. Lastly, *social* factors include weight stigma, bullying, influence of peers, and cultural idealizations [11, 15–19]. For example, loved ones (e.g., family and friends) commenting on physical appearance, dieting, frequent weigh-ins, or forced exercise, especially from mothers during adolescence, are all factors that increase the risk of DEEDs development [20].

Social media has also been identified as a significant contributor to the development of DEEDs. Dane and Bhatia's systematic review on social media use and body image among adolescents and found social media usage to be a strong risk factor for the development of DEEDs, poor body image, and overall mental health, with social comparison, thin/fit inspiration, and self-objectification as mediating pathways [21]. However, high social media literacy (i.e., the ability to create, evaluate, and analyze social media) and social media posts that emphasized body appreciation decreased the impact [21]. Thus, the more time one spends engaging with social media, the higher the chances of having body image and DE concerns [21].

DE is a broader category of skewed eating habits that evade Diagnostic and Statistical Manual of Mental Disorders, Fifth Edition (DSM-5) diagnostic criteria, including restrictive, emotional, or disinhibited eating, strict dieting, and concerns around controlling one's weight and shape through these behaviors [22–24]. However, it is essential to view DE on a spectrum of non-disordered eating to disordered eating and recognize that many of what people experience falls along that spectrum. DEEDs share similar attributes, such as individuals' negative relationship with food and their bodies, which may manifest in negative self-talk related to food, labeling food as "good" or "bad, "and engaging in diets, limiting, or over-consuming food [25].

They also may experience social isolation; negative, strong emotions; engage in physiological behaviors such as dieting; and psychological factors like perfectionism and obsessive thoughts [5, 15, 17]. However, where DE and EDs diverge is the effect on an individual's relationships, their daily activities, and the frequency of engaging in harmful behaviors [R Tilt, personal communication, 16 December 2020].

It is important to note that a person can have a harmful relationship with food and body image without having a specific ED. An example of this is Orthorexia Nervosa (ON), which involves an obsession with eating what society deems "healthy" (i.e., quality) without focusing on how much or how little one consumes (i.e., quantity) [27, 28]. Research on ON is limited, but this obsession can result in adverse health outcomes, such as malnutrition [27, 29]. While ON has similar attributes to those of DEEDs, it has yet to be classified as an ED within the DSM-5 [22].

The DSM-5 classifies EDs as a lasting disturbance of eating habits or behaviors that results in altered consumption of food and significant impairment of physical or psychological health and therefore constitutes behaviors that result in one having a classified ED [30]. There are various types of EDs, but for this study, the focus is on the most common types, which consist of Binge Eating Disorder (BED), Anorexia Nervosa (AN), Bulimia Nervosa (BN), and Other Specified Feeding or Eating Disorders (OSFED)/Eating Disorder Not Otherwise Specified [30].

BED involves individuals consuming ample amounts of food in a short period of time while simultaneously experiencing lack of control, shame, and guilt over three months, with an episode occurring at least once a week [1, 22]. AN is characterized by behaviors (e.g., overexercising, taking laxatives, ignoring hunger pains, etc.) and beliefs (e.g., distorted body image) that can result in a reduction of weight, restriction and obsession of food intake, and can result in lower heart rates and damaged immune systems [22, 30, 31]. BN involves similar binge episodes but is followed by purging behaviors such as vomiting or laxative use [22, 30, 32, 33].

OSFED/EDNOS refers to clinically significant disordered eating patterns that don’t meet full criteria for the above EDs [30]. Additionally, there is purging disorder, which involves individuals purging to control their weight but not consuming excessive amounts of food [30]. Though DEEDs can have a detrimental impact on individuals, families, and communities, those who experience DEEDs are often misunderstood. Many believe individuals are just going through "a phase" or that they have the "choice" to stop engaging in their DEEDs behaviors.

These notions are incorrect, and DEEDs can result in severe negative health outcomes and even premature mortality [1, 34]. One person dies every 52 min due to the severe effects of EDs, which are about 28 individuals each day [35]. EDs have one of the highest mortality rates of all mental illnesses, second only to the opioid crisis [1, 35]. However, though many are impacted, and some in a severe way, many who manage EDs tend to feel isolated and less connected to their loved ones [36]; otherwise known as feeling stigmatized. Stigma, in general, occurs when an individual's vulnerability (and the associated risk) increases, resulting in that individual being potentially viewed or perceived as viewed negatively by others [37]. Social isolation, which is often experienced with DEEDs and increases the risk of negative health outcomes, can be rooted in stigma due to fears of rejection, can impact their decision to share (or withhold) that information with others [36–38]. This can be problematic, as the internalization of negative societal perceptions can lead to adverse coping behaviors, increase stress, and reduce the willingness to disclose information to others [39]. To receive help from others, admitting one's DEEDs habits to oneself is only the first step. Eventually, individuals must disclose their concerns to trusted individuals, which is easier said than done. A study conducted in Australia by surveying 18- to 25-year-olds with various ED symptoms revealed that while there was a general belief that seeking help is beneficial, only a few participants with more severe symptoms of AN, BN, and BED believed they needed help.

The reasons for not disclosing were found to be concern for others, self-sufficiency, fear of losing control, denial, failure to perceive the severity of the illness, stigma, and shame [40]. These beliefs and assumptions held by others and the individual's experience may serve as a barrier to seeking help and hinder communication for those suffering from DEEDs. Additionally, the stigma of EDs may delay individuals seeking treatment. According to Hamilton and colleagues, ED patients delay care by an average of 5.28 years from the onset of their symptoms, which, for many, can be the difference between life and death [41]. Gilbert and colleagues discovered that the identity of the person and method of disclosure can have a significant impact on when and how people choose to disclose, with faster access to services being associated with disclosures that involve a healthcare provider, partner, or mother, further supporting the importance of raising awareness of DEEDs to not only those susceptible to the battle (i.e., disorder/disordered behaviors) but to those that can intervene and potentially prevent a fight EDs from ever occurring [42]. As one moves into later adolescence, peers become the focus of attention. They can increase or protect against the likelihood of not only DEEDs, but also, whether people feel heard, believed, or comfortable enough sharing that information with others to obtain the help needed.

Communication Privacy Management (CPM) is an interpersonal communication theory developed by Sandra Petronio to better understand how people manage the disclosure and concealment of their private information, as well as the guidelines (i.e., privacy rules) that influence who, when, where, how, and why personal information is shared [43]. Another major aspect of CPM is the idea of co-ownership. CPM suggests that when one shares private information, there is the assumption that it will be kept private. Therefore, the person receiving the information is the co-owner of that information [44]. This ownership is represented by metaphoric privacy boundaries that dictate how people protect their information.

CPM theory posits that dialectical tensions are at the center of privacy management. This means that we make decisions based on the need for both privacy (i.e., not disclosing) and openness (i.e., disclosing) simultaneously. In terms of openness, individuals may wish to disclose their personal information to "get it off their shoulders" (i.e., for cathartic reasons), for self-clarification, and to seek social support [45]. Additionally, individuals may believe that others should know this information about themselves (i.e., other-focused reasons) [46] and perceive it to feel closer to them (i.e., relationally focused reasons), such as increasing intimacy [45, 47].

On the other hand, revealing private information has potential downfalls such as conflict, criticism, hurting others, stigmatization, and relationship termination [38, 46, 48–50]. Many individuals also fear being isolated and viewed negatively by those close to them (e.g., friends, family, partners) after disclosing personal information [36, 49, 51–53], especially women, who tend to avoid disclosures and enact in the "role of concealment and secrecy" due to fear of judgment, negative emotions, and overall body concerns [49], p. 222]. As a result, individuals may feel more vulnerable/exposed/stigmatized [54] and therefore require more trusting, supportive relationships. The perceived quality of social interactions (i.e., high trust) notably impacts help-seeking behaviors, with lack of family support and understanding from peers has creating significant barriers for those contemplating disclosure [55, 56]. When individuals perceive that they lack an empathetic audience, the likelihood of feeling validated and encouraged to speak about their DEED experience diminishes, creating a cycle of silence and suffering [57]. This extends from family and friends to healthcare professionals, who listen and validate their patients. Healthcare professionals who understand and provide social support can enhance outcomes for individuals recovering from eating disorders [58, 59]. Such interactions support emotional well-being and educate individuals about the implications of their struggles, further empowering them to disclose their experience to others in their lives [56, 60]. This study aims to understand the social barriers and influences that contribute to individuals' decisions to disclose (or not) their DEEDs. As part of a larger study that explores this topic within the context of communication and public health, this paper focuses on the public health perspective. Furthermore, to explore and better understand the privacy rules (implicit and explicit), (non)disclosure around DEEDs, and the social and environmental factors that impact these decisions. Thus, the following questions were used to guide this part of the study:

- RQ 1: What are the factors that contribute to navigating privacy and disclosure related to DEEDs?
- RQ 2: How do the factors influence navigating privacy and disclosure related to DEEDs?

## Methods

This study aimed to understand the social barriers and influences that contribute to individuals choosing to disclose their DEEDs. The design of this study consisted of a semi-structured interview, developed using CPM [43], with additional questions tailored to address the research questions. Institutional Review Board (IRB) approval was sought prior to the start of the study. The interviews were conducted by the first author, virtually, and lasted between 40 min and an hour. To participate in the interview, participants had to be at least 18 years old, be able to speak, read, and write in English, and have experienced DEEDs. Participants were recruited through flyers posted in a large northeastern city and primarily on college campuses in the area. For participation to occur, participants had to scan the QR code on the flyer and sign up via a short Qualtrics survey, in which they answered questions to determine their eligibility and provided an email address to set up the interview. If participants were eligible, the first author contacted them via email to schedule a meeting via Zoom, using the email address they had provided.

Participants (*n* = 15) consisted of individuals aged 18 and above who reside in the northeastern part of the United States. No additional demographic information was collected to maintain anonymity. The interview was recorded via Zoom with the participants' permission. Only the auto-generated Zoom transcript following a recording and the interview audio were saved. These were reviewed in tandem during the transcription process to ensure the interview was captured accurately.

A thematic analysis [61] was conducted to identify study themes. This process began with all researchers conducting an independent review of interview transcripts (*n* = 15) (totaling about 740 min of audio and 637 pages of text) to immerse themselves in the data and to take preliminary notes on patterns presented that best help to answer the research questions posed. Next, the researchers met to discuss their notes and finalized emergent themes found within the transcripts with the use of cross-coding among all the researchers listed on this study. The themes were finalized based on a couple of factors: 1) how much the themes helped to answer the proposed research questions, and 2) how they reflected the impact on public health, since that was the focus of this study. Then, researchers reviewed the transcripts once more to highlight specific examples/quotes that reflected each theme. The first author reviewed all these themes and examples once more to complete the overarching themes and examples from the transcripts presented in the results.

## Results

Five key themes emerged from the qualitative thematic analysis, revealing the nuanced and interconnected ways individuals navigate privacy and disclosure related to DEEDs. These themes include (1) *family influence and comments,* (2) *social media influence*, (3) *influence of healthcare*, (4) *justification factors*, and (5) *comorbidities*. These themes highlight the complex web of societal expectations, personal motivations, and interpersonal dynamics that influence when, how, and to whom individuals choose to disclose their struggles. Taken together, these insights shed light on the multifaceted privacy rules participants create and recalibrate as they manage stigma, seek support, or attempt to maintain a sense of autonomy in the face of DEEDs. These themes are discussed below, accompanied by quotes from participants to illustrate them.

### Family influence and comments

Family influence was the most dominant theme in participants' discussions, "*shap(ing) like my idea of food and like, diet culture and society shaped this in this way" (Interviewee 5).*

Many described growing up in households where body size and food choices were scrutinized,often leading to complicated relationships with eating and self-perception. Parental influence, particularly comments from mothers, fathers, and extended family members, contributed to feelings of shame, restriction, and internalized food rules that many grappled with throughout adolescence and even through adulthood. For some, parental body talk, and diet culture messages became deeply ingrained. One participant recalled how their mother's insecurities were projected onto them, particularly during childhood moments, such as shopping for clothes.

*She projected a lot of her feelings about herself onto me when I was younger. At the time,I didn't think much about it, but over time, I realized those comments really affected me. If something didn't fit, it wasn't just "Oh, let's get a different size." It was "This is your usual size, and you're supposed to always fit in this. And the reason why you're not fitting in it is because you've been eating this and that." (Interviewee 12).*

This internalization of body surveillance often started early and was reinforced repeatedly throughout one's lifetime. Mothers were often described not only as enforcers of food-related rules but also as navigating their complex relationship with eating. This dual role often sent conflicting messages to participants, leaving them uncertain about how to view food and their own health-related needs, as mothers usually contributed to a toxic mindset around food.

*And a lot of it was like my mom would talk really badly about just like food and stuff and what not but then like because my parents divorced at an early age, she would like try and help and give us treats and like help us feel better. Like she would lecture me, being like "oh don't eat all that" and like "you need to eat this, you need to eat that", but she would like let me have my moments of like, I don't know. It was so weird because it's like "no you can't eat this blah blah blah”, and she was like "I bought you ice cream!" (Interviewee 5).*

Some mothers framed their concern around food as protective or health-focused rather than critical, yet the impact was still one of heightened monitoring and self-consciousness.

*And again, I don't think she was like, she was never like making comments about like, "I think you look bad," like all of this stuff. It was all like coming like "I would be worried about how I look." (Interviewee 15)*

On the other hand, some mothers tried to provide support for their children struggling with DEEDs. However, their attempts were often filtered through lenses of comparison or misunderstanding, especially when siblings were involved. In some cases, a sibling's athleticism or body type changed how concern was expressed or received.

*I think my mom kind of thought she was like helping me like, or both me and my sister, but my sister, was a division one athlete, so she was still like very much playing sports, and so is a little different. (Interviewee 15)*

For other participants, family members' body shaming extended beyond their parents, with grandparents reinforcing negative stereotypes as well. Some even described multigenerational attitudes toward dieting, where food restriction and weight monitoring were normalized, "*and my grandma would kind of like make fun of my mom sometimes during dinners, like "what can't you eat now?" like "what diet are you on now?" like stuff like that" (Interviewee 12).* Another participant mentioned how their grandma would even make targeted comments about individuals in a matter-of-fact way, "*and like say, 'they gained weight', yeah, or 'they lost weight'" (Interviewee 15),* comments this interviewee indicated their friends would never say nowadays due to the stigma it perpetuates around body image. Fathers also played a significant role in shaping body image through indirect but impactful comments that tied athletic performance to body size. One participant recalled a remark from their father that stuck with them for years.

*He made this comment 8 years ago, and I still remember it. "Oh, don't you ever like look at the girls on the team, and just like, think kind of like, 'oh, well, yeah, they're doing better because they're skinnier and they're built for the sport." And I was like "Whoa there! Yes, I do look at them. But, like, I don't need you pointing that out to me and telling me that I should be considering that." (Interviewee 12)*

Many participants indicated growing up in athletic or fitness-focused families and how food and weight were constantly discussed in their households, like "*oh, she looks horrible" or "oh, he looks so overweight" (Interviewee 3),* making them hyperaware of their bodies from a young age and causing them to feel intense pressure to conform. This environment often sets the stage for unhealthy comparisons and DE.

Beyond individual comments, food control (i.e., which, when, and how much food) within the household shaped long-term eating habits. Some participants recalled growing up in environments where certain foods, especially sweets or "bad foods," foods that one does not deem healthy, were strictly regulated, reinforcing moral behaviors toward eating, where participants tended to form restrictive patterns around these "forbidden" items.

*And I think the reason I can't keep sugar in my house is because neither of my parents could. Like, it was never in my house growing up because if the adults can't keep it, then there's no way the children could either. But that was something I've grown up with. Like, there's never any sweets in the house at all. I got really good at making mug cakes. (Interviewee 1)*

Participants from immigrant families described an added layer of stigma. One shared that mental health and DEEDs were rarely discussed, making it difficult to seek support or treatment.

Disclosure felt risky, not only because of fear of judgment but also because it could trigger parental ridicule. Several participants, thus, avoided confiding in their parents out of concern that their struggles would be interpreted as a failure of their upbringing.

*I come from, like, a family, like, both of my parents are immigrants, and, like, this whole, like, idea of, like, struggling with something like that is not, like, something that's very accepted. (Interviewee 3).*

### Social media influence

Social media emerged as another significant influence in shaping participants' relationships with food, body image, and disclosure decisions. For many, online platforms served as both a mirror and a megaphone, reflecting societal beauty standards while simultaneously amplifying disordered messages. Participants described early exposure to content that glamorized DEEDs behaviors, particularly within niche online communities. One participant recalled being drawn into the world of "*eating disorder Tumblr"* (Interviewee 5), knowing it was problematic but feeling captivated by the romanticized aesthetics and idealized thinness it promoted. Another participant added, "*I always, like, compare myself to, like, the girls on Yeah. Pinterest or Instagram, things like that, or YouTube" (Interviewee 4).*

Others noted how algorithmic patterns reinforce harmful content. One participant explained that managing a work-related account caused their feed to become saturated with diet and fitness content, creating a feedback loop that was difficult to escape.

*The work account that I do social media for, their content got more curated towards, like, eating and diet, and workout. So, I was like, oh, this is bad. It's on multiple of my accounts. So, you're just being constantly bombarded with it. And it's like and then, of course, once they have the algorithm, it's like, once you're looking at it, then it's hard." (Interviewee 1).*

Another interviewee observed how influencers on TikTok normalized restrictive eating through trends like "What I Eat in a Day" videos, often showcasing unrealistic and minimal food intake, making restrictive eating appear aspirational even "*I feel like there is a shift in the culture nowadays and like in the past summer. There are a lot of influencers on Tiktok where it's just like what I eat in a day, and it's a very, very small amount" (Interviewee 8).* This constant social media exposure can contribute to feelings of inadequacy and internalized comparison that can lead to some DE behaviors.

Participants also expressed concern about how social media eroded emotional intimacy and fostered a culture of apathy, ultimately discouraging vulnerability. One noted that when she talked to friends, *"my problems and things, it didn't exactly feel like they cared or were, like, genuinely sitting down to listen. It was like, I don't know, it was kind of dismissed" (Interviewee 5).* In other words, many were listening to respond rather than truly understanding what individuals were going through. This perceived lack of genuine concern was attributed in part to the oversharing culture and desensitization that occur on digital platforms, where vulnerability is common, but empathy is scarce. Thus, when it comes to important topics such as eating and body image concerns, this lack of empathy and understanding can create isolation in which.

DEEDs thrive. Several participants also described how social media fed into their sense of not being "sick enough" or not fitting the stereotypical profile of someone with an ED, "thin, white, middle-class girls," which made others feel hesitant to disclose, unsure if their experiences would be taken seriously or seen as valid.

*I don't think it's a rule, but I definitely, like, do see that a function that it's, like, a certain type of, like, usually thin, like, middle class, like, white girl who is most likely to have these problems. I guess, yeah, that's, like, like, I fit that description too. So, yeah, I don't wanna, like, center that always or, like, make it seem like it's you know, that my disclosure of, like, something that was hard is, like, any worse or, like, harder than anyone else's childhood difficulties, yeah, for lack of a better term. (Interviewee 2).*

The aestheticization of bodies, where trends move from glorifying ultra-thinness to so-called inclusivity and back again, also added to participants' confusion and anxiety. One participant likened this to the evolving narrative of the Victoria's Secret fashion show.

*Like, they made it more inclusive because people asked for them to be more inclusive. But then they got like so much backlash about it, and they're just like, "Oh, I don't want to see like models and bigger bodies, because, like that ruins the fantasy." So, then they had to stop it. But now they're bringing it back, and they're just like it's going to be how it used to be. And now, like everybody's really excited for it, even though they asked for it to be more inclusive in the first place. (Interviewee 8).*

In other words, this participant feels that bodies are trends that go in and out of style like clothing. Another participant also described how many influencers promote unhealthy or "detox" eating habits. This is particularly harmful to teens, as it normalizes unhealthy eating habits and disseminates false information about healthy eating and exercise.

*I think, obviously, like social media plays a huge role in it because of what I think. That's mainly how this like diet culture is kind of like perpetuated in general. And I think a lot of it, too, is like people masking or like their diet, whatever, under something else, like like clean eating, or like trying to like detox, or whatever is. And I feel pretty thankful that I think I'm able to just see through that. I don't watch it. If I see it on my, on any of my social media. It's just like the not interested thing and then move on. But I think a lot of people like aren't as aware of the fact that these behaviors that certain influencers, or whatever creators are exhibiting aren't like detoxing. It's just not like a healthy way of having a relationship with your body, with exercise, with whatever. (Interviewee 15).*

Online bullying related to body size emerged as another prevalent part of this theme. A participant observed that such bullying not only intensifies stigma surrounding body size but also highlights how the anonymity of the internet can be exploited to spread hate and derogatory remarks about people's bodies: "*I think there are people on the internet that are just like awful.*

*Obviously, that just like are commenting on other people's bodies all the time and like they just they know they're being rude and mean, and all that" (Interviewee 15).* On the other hand, the same participant noted how social media can be used to promote body positivity and combat the harmful stereotypes often promoted online. Further, social media can be used to share experiences with DEEDs and decrease stigma.

*Overall transparency of like, especially Gen. Z. And they're like mental health struggles has been super helpful for this moment, but at the same time like, and I think social media has helped that a lot as well. So, I think I think it's helpful in some ways, and then really, obviously harmful on others. And that's like, that's that's not a new conversation. Obviously but I also do think that again, like there are also creators, influencers, whatever you want to call them, that are on their way to combat these things, and like calling out some of these bad like, not good behaviors for what they are, which I think is also awesome. So, I don't know. I think the diet culture thing again, as I say, like. I think it's super different, based on generation. I think social media has really brought to light that like, especially like Gen. X. (Interviewee 15).*

### Influence of healthcare

Healthcare interactions played a crucial role in shaping participants' privacy rules surrounding DEEDs. While these environments are often expected to offer support and guidance, many participants described experiences that were dismissive, demeaning, or detached. Therefore, rather than fostering engaging, empathetic, and egalitarian interactions, these encounters often reinforced silence, secrecy, and seclusion.

Several participants shared not feeling believed or taken seriously by their healthcare providers. One participant emphasized how the lack of trust and validation from their provider was a core reason they withheld information about their DEEDs.

*"I'd say the thing that I always like to bring up when I like tell people about. This is like the role that healthcare and not being believed by my healthcare provider played in this" (Interviewee 6). While another participant discussed feeling their provider only viewed it as something that could be changed with weight gain, "they [pediatrician] were handling it more as just like I was underweight and not like it was a, I guess, like a mental disorder" (Interviewee 10).*

Routine doctor visits were also described as uncomfortable and undermining, contributing to internalized stigma for some. One described how being repeatedly labeled as "slightly overweight" shaped their perception of their body, despite knowing internally that the label did not accurately reflect their reality, "*like every time at the doctors [PCP}, I would be like for my annual, just like slightly overweight, or something, and like over time that then meant something to me, and, like, I would know, like at the doctors like that's not correct. That's not the correct one. I know what I am" (Interviewee 12).* As a result, the way their weight was discussed during health visits contributed to internalized stigma and distorted self-image.

Health professionals in fields such as **mental health** (i.e., known to be more knowledgeable of emotional well-being) also often failed to provide a safe space for some to disclose their personal experiences. Participants recounted instances of judgmental responses that undermined trust, causing them to retreat from further engagement.

*My therapist kind of she wasn't very good like I would describe certain behaviors that would be very clearly like representing depression, like I think I hit a period where I just like couldn't like get the will to like shower for like a couple of days at a time. And she was like, oh, that's disgusting. I was like, okay, so I'm never talking to you about this again, she left the clinic, they were like, “do you want someone new” and I was like,“no, I'm good.” (Interviewee 12).*

In addition to negative interpersonal experiences, systemic barriers further compounded participants' reluctance to disclose their experiences. For instance, another significant barrier participants highlighted was the issue of access to *Healthcare, like quality mental health services. Especially because of our horrible private insurance market in the United States, it's super hard to find people that are in network, that are specialized in disordered eating, that don't have ridiculous wait times* (Interviewee 2).

Therefore, the asynchrony in healthcare delivery (e.g., long waiting times), lack of access to quality services, and exorbitant cost of treatment (i.e., often not covered by insurance), deter many from not only disclosing about their experiences but also seeking the care needed.

These logistical and systemic barriers intersected with perceptions of time scarcity during appointments, making such concerns feel secondary or unimportant. For example, participants also described feeling unable to prioritize their mental health in routine medical visits due to time constraints and the need to address more immediate concerns.

*I would feel uncomfortable talking about this in a professional setting, and I probably wouldn't bring it up at the doctor's office unless I was asked, do you have a history of x y z? I feel like it's not, like a concern that's, that has, like, a lot of impact thankfully on my day-to-day life. Yeah. Most of the time when I'm at the doctor, I'm like, trying to remember, like, okay, I need this prescription refill, then yeah. I feel like it, it's so hard to get physical, with the wait time this year that I'm just like, yeah. Moment of time, like, let's go through the list. Let's go through, like, the things that I think are the most pressing. (Interviewee 2).*

Still, some participants recognized that awareness around inclusivity in DEEDs has improved, even if access to care stays limited

*"I think I've seen a shift, which is positive about inclusivity. You know, it can happen to anyone regards of race, socioeconomic status, gender identity, etc." (Interviewee 2).*

### Justification factors

Stigma surrounding DEEDs often resulted in individuals engaging in justification factors to rationalize or conceal their behaviors. Participants often described sports, exercise, and demanding schedules as rationalizations for their DEEDs behaviors. These contexts allowed DEED patterns to go unnoticed or be excused as discipline. Because athleticism is often associated with health and success, participants were able to justify restrictive behaviors under the guise of being active or busy, avoiding scrutiny from others and even from themselves. For example, a participant mentioned, *"while my friends are playing in that like playground, I would just, like, walk around it just, like exercise, and it began from there"* (***Interviewee 4***). As a result, the normalization of their addictive activity from an early age can be seen as a form of justification for their experiences with DEEDs. Several participants grew up in highly active households or were immersed in fitness- centered environments. In these contexts, excessive physical activity was rarely questioned and often encouraged, blurring the line between health promotion and harmful overexertion. As one participant explained:

*I really started with over-exercise, because I think that it's something that was like it was never questioned in my family like to be working out was like a good thing. My sister is an athlete. My parents were athletes like they, you know. That was just kind of the norm for us, and they thought it would make me better at my sport. And I guess there's some validity in that. But I think in the beginning I got away with like a lot of over-exercise because of that. (Interviewee 6).*

In many cases, such as with interviewee 6, it even offered a socially acceptable way, via sports and "staying in shape", to mask disordered patterns. Additionally, being part of competitive sports, which fostered environments where thinness was the norm and constant body surveillance, further reinforced these behaviors:

*I was always like, pretty active, as a kid. Like, I've been dancing for most of my life and then I've been like running and just like always on the move. So, I was never just like did nothing and sat around, so most distance runners are pretty like thin, lanky. (Interviewee12).*

Participants, such as interviewee 12, described how environments like distance running and dance emphasized thinness and body surveillance, resulting in justifications for certain DEEDs behaviors to flourish. Not only this, but many sports, such as dance and cheerleading, also highly value aesthetics, which can result in individuals engaging in DEED behaviors to fit the image or look that is required for the sport. In such competitive athletic cultures, tracking food intake and body metrics became routine, making it easier to justify disordered habits. Some participants even described documenting their physical progress through photos.

"*I would take like progress photos, like check to see how I'm looking before and after going for my run that I had for that day" (Interviewee 12).* This example from interviewee 12 and even other participants highlights how some may justify concerning behaviors, possibly leading to DEED behaviors, by framing them as efforts to improve as an athlete. Fear of disrupting physical performance also became a justification for meal skipping,

*I would never eat during practice or like before. I had practice, even though, like I usually had like 30 min or so. But I was also like really like kind of afraid of like throwing up like, you know, I just had something, and then I go and run. So, I'm like, I'm not, I'm not about that. So, I would just kind of like fend off (Interviewee 12).*

In other words, this participant used performance concerns to mask their restrictions and justify their concerning behaviors that could result in DEED behaviors. In some cases, exercise and thinness were interpreted as symbols of self-discipline, ambition, or control, especially when seen through the lens of societal assumptions about fitness and health. Several participants reflected on how these ideals allowed them to justify their behaviors while reinforcing internalized pressure to maintain a certain image.

*I think one of the big things has to do with exercise, because when they see someone who's thin, you automatically assume that they're healthy, and you like place a lot of ideals on them like, oh, they must work out a lot, which means that they're super disciplined, which means that they're ambitious, and they like know how to chase their goals, and they like say no to things like cakes, and like ice creams and things like that. (Interviewee 8).*

These assumptions offered participants added protection from scrutiny and were a way for them to justify that their behaviors are not, indeed, concerning.

In addition to athletic justifications, structured daily routines such as demanding academic or extracurricular schedules further enabled meal-skipping behaviors to go unnoticed and without intervention. Long days with little oversight allowed participants to skip meals or avoid eating around others, for example,

*I had a very demanding schedule like I would get up. I would leave for school at 6:30 in the morning. Be there through classes until like 3 o'clock. Have practice from 4 to 6. Don't get home till 7 or 8 o'clock. So, I'm like at school or at practice all day long, and my school also, like your schedule, was packed. There were times when you didn't have a lunch period. You would have to eat lunch during class. So, it either be like, bring something, or they let you go to the cafeteria to get something and bring it up to class so you can have it. And then so with that, as it's like going, I realize, like, oh, I'm not my mom's not like watching me, she doesn't know like what's going on. All my friends have different lunch schedules, too, so like, I knew if I didn't have something then my friends wouldn't notice it. (Interviewee 12).*

As described by the interviewee, restrictive and busy schedules helped to conceal DEEDs from parents or caregivers, as their lack of physical presence during meals reduced opportunities for others to detect concerns.

Other participants noted that major life transitions, such as starting college, further exacerbated body image concerns and normalized unhealthy behaviors. With fewer external structures, many described increased fixation on food and exercise as coping mechanisms for uncertainty or social comparison.

*I think, also, just like being in a freshman in college, like so many young women like have really bad like relationships with their body, with food, with exercise, with everything, I was like very hyper fixated on it, and then I think just like being a freshman in college and stuff like that, and like on top of the fact that there was literally nothing going on like there's nothing going on. So, there was just not really much else to be focused on other than like school, and that so I think that played a pretty big part in it as well. (Interviewee 15).*

As interviewee 15 noted, seeing other individuals struggling with exercise and food justified DEED behaviors. They further described how the transition to college was difficult in shaping their relationship with their body, food, and exercise.

Finally, a prominent misconception highlighted by participants was that DEEDs primarily affect women. This belief served as justification for many men to dismiss or downplay disordered behaviors – both in themselves and in their peers. Participants reported frustration at the normalization of concerning habits among male friends and the invisibility of DEED experiences in male populations.

*I've also seen, like some pretty, pretty, unhealthy behaviors with food, with some of the guys. My age I will say that like that is something that is super concerning, because they don't think it's an issue. None of them are aware that this is even a thing they pretty much think it's only a girl thing, and [don't want to be affiliated with it]. I've talked to my boyfriend about it a lot. because there were times where I noticed, like at least some of his roommates, and I was like that whatever your friend is doing. I'm just letting you know that it doesn't seem healthy at all, and it's like, Oh, no, they're fine, they're fine, they're fine, they're fine. And I'm like, okay, I'm not going to get too much into this like your friend, whatever. If you think it's fine, it's fine, but more concerning to me, because obviously like this can happen to young men as well. And they I just don't think they acknowledge that it's even a thing." (Interviewee 15).*

As stated by interviewee 15, this belief that DEEDs cannot impact men displays this justification that many who identify as male tend to you use for any behaviors they may engage in that are or can be concerning. In other words, there is this general lack of awareness and the presence of stigma surrounding DEEDs in men, which in turn prevents them from not only disclosing their experiences but also seeking the help they may need. This gendered assumption not only reinforces stigma but also contributes to a serious gap in prevention, early detection, and support for men struggling with DEEDs.

### Comorbidities

Many participants described how their experiences with DEEDs were deeply entangled with other mental health challenges. Anxiety, panic disorder, obsessive–compulsive disorder (OCD), and even substance use was often present alongside or even prior to the development of DEED behaviors. These comorbidities often intensified participants' negative relationships with food and body image, while also influencing the privacy rules surrounding disclosure. Within the framework of CPM, these overlapping challenges may serve as both catalysts and inhibitors to disclosure, altering privacy boundaries based on emotional need, risk–benefit analysis, and trust. For some, seeking care for mental health concerns became the indirect gateway to recognizing DEEDs, while for others, these overlapping conditions compounded feelings of loss of control, shame, or isolation. One participant shared that they initially sought support not for DEEDs, but for anxiety and panic disorder

*"but at first it was only for just my like I had, I have anxiety and panic disorder. So, I went for that" (Interviewee 12), while another participant "ended up like going to a psychiatrist, and I got prescribed antidepressants for my anxiety, which was kind of a separate thing" (Interviewee 15)*

These accounts highlight how psychiatric symptoms often served as the initial entry point into care, even as underlying DE remained unaddressed.

Participants also highlighted the emotional and behavioral links between anxiety and DEEDs. For some, food and body control became maladaptive strategies for coping with internal distress:

*"I do struggle with a bit of anxiety, and the eating thing that comes with it" (Interviewee 7).*

In some cases, participants reported that depression symptoms made their DEED worse, particularly during times of social isolation or lack of external accountability. Several described periods of being alone, such as while away on co-op or study abroad, as catalysts for worsening symptoms.

*I had suffered from some depression symptoms before, and they were definitely coming back. And I was basically like I was completely alone. So, I basically had free, free reign to kind of like just let my eating disorder go wild. (Interviewee 6).*

In other cases, obsessive–compulsive tendencies, particularly those related to food, health, and body monitoring, were also prominent. Participants described strict routines around eating and exercise as initially goal-oriented, but ultimately compulsive and distressing. One participant noted how strict rules around eating and undiagnosed OCD fueled their DE behaviors.

*"so, like so then I started, like, being more particular about what I was eating, and then it kinda spiraled from there. I also struggle with OCD. It's something I've struggled with my whole life, but never, like, received help with" (Interviewee 3)*

These overlapping patterns often intensified distress and created a cycle that was difficult to break, highlighting the complex interplay between general obsessive–compulsive tendencies and food-specific anxieties. Further, the delayed diagnosis and management of comorbid conditions, such as OCD, contributed to prolonged distress and a lack of insight into underlying DEED behaviors, "*also, I wasn't diagnosed with my OCD, so a lot of that I wasn't medicated yet. Things are a lot better now that I'm on medication" (Interviewee 11).* This delay in diagnosis may contribute to prolonged engagement in maladaptive coping strategies like restriction or overexercising, creating a self-reinforcing cycle of symptom management through food control.

Substance use also emerged as a contributing factor. In some cases, participants used substances alongside DEED behaviors or in response to similar internal stressors. The early onset of these behaviors and their physical consequences contributed to body image concerns and accelerated the progression of DE trajectories. One participant described early onset use:

*It started when I was 13. I got into the substance use very, very early on in age. So, when I was 8, I started using a lot of like smoking and drinking. I started thin, and by the time I got into 13 I started to see that because those things have a weird effect on your body, because, like, you know, your body starts to grow before puberty. (Interviewee 7).*

In other words, highlighting how early exposure to substances may have contributed to body changes and body image distress, further compounding the risk for DEEDs.

## Discussion

Findings from this study highlight the complex social, cultural, and structural factors that shape privacy management, as well as the development and disclosure of DEEDs. Family influence, social media exposure, impact of the healthcare field, justification through sports and work, comorbidities, and overall societal expectations all play significant roles in how individuals navigate their relationships with food and body image. These findings support previous research indicating that these factors play a role in the development and continued DEED behaviors [62–66]. Our study enforced and expanded previous findings on the familial influence of DEED development. We found that family environments significantly shaped participants' privacy rules and justifications for DEED-related behaviors. Many participants highlighted harmful comments about eating, shape, weight, and appearance from family members as significant influences for DEEDs. Our findings are consistent with other studies that have analyzed the impact of adverse, non-abusive, familial life experiences and DEEDs, specifically through comments. For example, a systematic review by Grogan and colleagues revealed that family comments significantly impacted those with BN and BED compared to psychiatric controls. Familial teasing from parents and siblings were found to increase the risk of DE directly and indirectly through the influence of their teasing on poor social functioning and negative self-esteem. This teasing also increased sensitivity to comments and anxiety over time [67]. Furthermore, this represents how rules core criteria such as cultural expectations and family norms hinder disclosure about disordered eating. Within the framework of CPM, such normative pressures inform the implicit rules individuals use to determine what information is shareable and with whom. In this case, the fear of judgement, misunderstanding, or additional emotional harm from family members led participants to enact stricter privacy boundaries around their DEED behaviors. Another study surveyed a representative mixed-sex sample of 2204 Australian adolescents (12–19 years) and found that perceived negative parental comments were significantly associated with DEEDs and psychological distress in both sons and daughters, while perceived positive parental comments on shape/weight were significantly associated with lower psychological distress and DEEDs for daughters only [68]. These results mostly remained when controlling for factors known to be strongly associated with DEEDS (i.e., BMI percentile) and psychological distress [68, 69]. Although the population in Dahill and colleagues’ study was younger than in our study (18 years and older), our findings provide context for how parental comments can have a lasting impact on individuals. Additionally, our study provides an opportunity to understand better how familial influence impacts not only past and present DEEDs but also the forms of support that are detrimental and beneficial to the prevention and intervention of DEEDs. From a CPM perspective, these findings illustrate how ongoing familial commentary becomes internalized, influencing not only self-perception but also the management of private information. Participants often described a calculated silence of choosing not to share their struggles to avoid emotional exposure, conflict, or further invalidation. As such, family dynamics not only contributed to the onset and maintenance of DEEDs but also shaped the emotional and relational triggers, catalysts, that governed disclosure decisions [26, 74]. The chronic invalidation some participants experienced led to privacy boundaries that were not just protective, but isolating. Findings from our study also further support and supplement previous work on the influence of social media on DEEDs. Social media tends to reinforce body ideals and glamorize.

DEED behaviors through trends such as "What I Eat in a Day" videos or "Fitspo" content. It is important to note that while social media contributes to the normalization of DEED behaviors, it is not the sole cause. Exposure to appearance-focused platforms such as Instagram, for example, has been shown to have a positive correlation with body dissatisfaction and DE, primarily through social comparisons [70]. Participants in our study recalled early and prolonged exposure to content that glamorized restrictions, especially within niche online communities such as "eating disorder Tumblr." Through the lens of CPM theory, these exposures served as catalyst criteria that shifted privacy boundaries, normalized restrictions, and decreased the perceived need for disclosure. This catalyst altered the rules they used to manage private information, such as how, when, and to whom they disclosed their behaviors, and fostered greater internalization of harmful norms [26, 74]. In some cases, the normalization was no longer seen as private or unusual enough to warrant conversation.

Higher levels of social media engagement, specifically with highly visual social media (i.e., frequency and volume of posts) like Instagram and Snapchat, have been associated with an increased likelihood of experiencing DEEDs among adolescents [71, 72]. Additionally, CPM theory helps to frame how these visual exposures impact individuals’ privacy management rules by making comparisons more personal and salient. For example, social media-based appearance comparisons, compared to no comparison, predicted a decrease in body satisfaction and an increase in urges to restrict food intake, exercise, and overeating [71, 72]. The findings from quantitative studies and narrative elements in qualitative research offer a holistic view of DEED development and disclosure. This study provides context on the multitude of intersecting factors that contribute to DEEDs and adds context to the impact of social media on DEEDs. Our participants' experiences align and add to previous research, reinforcing that many young individuals with DEED symptoms refrain from seeking healthcare due to barriers like poor mental health literacy, stigma, and negative experiences with healthcare providers who lack adequate knowledge or fail to recognize EDs [73, 74]. Healthcare providers and systems play a vital role in the disclosure of DEEDs, as they often hold the key to connecting patients with resources and often rarely provide a space that patients feel safe enough to disclose. Past research supports this within a pediatric setting through which patients experienced weight bias that resulted in exacerbating the risk of DE behaviors, including restriction, purging, and bingeing, and an overall lack of trust in their medical care [74, 75]. Participants in this study reported feeling dismissed or overlooked when brave enough to disclose. For others, the provider comments about being overweight, shaped body perception, and distorted self-image. As patients navigate privacy boundaries in the healthcare setting, providers must validate patients’ experiences, minimize weight-stigmatizing language, and support body neutrality [76] On the other hand, one participant articulated that being treated as "just underweight,"rather than having a mental disorder diminished the impact of the issue. These experiences extended into visits with mental health professionals as well, further limiting safe spaces for disclosure and perpetuating stigma around DEEDs. One negative experience in healthcare can dissuade people from seeking out or adhering to treatments, which are a crucial part of the recovery process. Our study provides firsthand accounts into how healthcare systems and providers intersect to shape participants' privacy rules. While one participant noted awareness of DEEDs in all populations has improved, most of our participants disclosed the negative influences healthcare systems and providers had on their DEED disclosure and development. Previous qualitative research indicates that being recognized and supported by empathetic professionals and having opportunities to discuss body image and shame in a safe environment are associated with greater hope and movement toward recovery [77, 78]. Providers should not bear the entire responsibility for healthcare’s impact on DEEDs, especially as systemic restraints like the limited time of visits, lack of structural support and training, and insufficient processes to facilitate DEED conversations [79]. As highlighted by multiple participants, systemic barriers like access to mental health services may dissuade individuals from seeking care. Our findings, along with those from previous research, underscore the need for healthcare systems to adopt patient-centered, stigma-free approaches, enhance provider education, and establish supportive pathways for early detection and intervention for all forms of EDs [77, 78, 80].

However, there are many positive and improving healthcare practices that encourage and support disclosure. Providers may choose to self-disclose to facilitate disclosure and foster trust within their relationship [81]. As the research and understanding of DEEDs evolves, so must the recommended treatments to incorporate more individualized and culturally competent care.

Research across the last decade has highlighted significant genetic components and environmental factors that individually and combined contribute to ED risk and development that should be included in provider-patient conversations and screenings [82]. Positive shifts in DEED treatment includes the use of multi-disciplinary teams (typically consisting of a medial practitioner, mental health professional, and dietitian) that collaborate with patients and their support systems to holistically provide care and partner with patients throughout the process [82].

Additionally, innovative technological solutions, or internet-based interventions, may help reduce the stigma associated with disclosure. Rohrbach et al., conducted a randomized control trial and found internet-based self-help and expert support both individually and combined significantly reduced DEED symptoms compared to the control group (i.e., those who received usual care) [83]. Furthermore, there are associations such as the Association for Weight and Size.

Inclusive Medicine (AWSIM) that aim to educate, mentor, and foster community for health professionals with the goal of fostering a more inclusive medical and health system. The current study reinforces and expands previous research through the incorporation of systemic and interpersonal healthcare barriers that contribute to DEED development and disclosure. Findings from our study also convey how DEEDs coincide with other mental health conditions, emphasizing that DEEDs are rarely singular issues. Instead, they reflect complex, overlapping psychological burdens that often go unrecognized or unaddressed. Comorbidities, specifically anxiety, panic disorder, OCD, and substance use, for example, are intertwined with DEED development, with anxiety, mood disorders, and substance use disorders affecting up to 62%, 54%, and 27% of individuals, respectively [32, 84]. Many of our participants disclosed experiencing other mental health conditions that influenced DEED development or exacerbated symptoms, highlighting specific aspects of the other struggles that connected to their DEED.

Previous large-scale studies and systematic reviews indicate individuals with ED's experience higher rates of anxiety disorders [85, 86]. These results are affirmed by cohort studies that revealed more than half of their participants with AN experienced Major Depressive Disorder (MDD) in their lifetime, with a third experiencing generalized anxiety disorder or social phobias, and a quarter being diagnosed with OCD [87, 88]. As our participants recounted, these comorbidities can encourage or hinder disclosure and could even lead one to being diagnosed with DEEDs without any prior suspicion. For some of our interviewees, their other mental health conditions were the reasons they initially sought professional help. With treatment, their DEEDs may have improved as well, even if they stayed unaddressed. Previous research also indicates that they can exacerbate psychological distress, increase feelings of shame, and heighten fear of stigma, all of which can make individuals more reluctant to disclose their ED symptoms or seek help [32, 84, 88]. Our study highlights the interplay between DEEDs, comorbidities, and disclosure through the lens of personal experiences. Through this approach, we are able to uncover and further explore the motivations and barriers for DEED development and disclosure, using theories such as CPM.

The theory of CPM, as it relates to stigmatized health topics such as DEEDs, is also reflected in our findings. CPM offers a valuable framework for understanding the complex relationship between privacy and disclosure, particularly in contexts where individuals must determine when, what, or how much of their private health information they wish to share [89].

This theory emphasizes that people create privacy rules that dictate disclosure or nondisclosure [89]. However, privacy rules are prone to change. While core criteria are more durable, catalyst criteria account for when there is a need to change privacy rules [89]. Such times include changes in motivation or when situations change, and therefore, different privacy rules are needed [89]. In the case of DEEDs, stigma, shame, and cultural/family norms may heavily influence the decision to withhold information, while trust or emotional distress may motivate disclosure. Our findings align with these tenets of CPM, highlighting how individuals weigh risks and benefits when disclosing their experiences with DEEDs.

There are several limitations to consider when interpreting the findings of this study. As with most qualitative research, the findings of this study are not intended to be generalizable [90, 91]. The sample of individuals (*n* = 15) in this study does shed light on DEED issues, experiences, perspectives, and associated factors. However, these interviews are but a small, specific group of people and as such, may not capture the full breadth of experiences by individuals who have engaged in DEEDs behavior. Additionally, since we posted flyers within a college campus and city, we want to acknowledge that it introduces biases toward younger, more educated populations. We acknowledge that the lack of diverse individuals in this study is essential to understanding how DEEDs may impact other populations. However, as this is a qualitative study, the intent is not to generalize the findings in this study to other populations, but rather to gain insight into the experiences of those with DEEDs. Future studies should consider using other methodologies to understand how different populations experience DEEDs. This information is still helpful in developing interventions to address DEEDs. Second, given the sensitive nature of DEEDs, some participants may have felt reluctant to share their experiences fully. Additionally, some participants may have had difficulty recalling or accurately articulating past experiences with DEEDs, especially if those experiences occurred several years ago or if they are currently in recovery. Third, thematic analysis reflects the researchers' interpretations of the data and participants' responses, which are shaped by our positionalities and assumptions. Although reflexivity was practiced, other researchers conducting the same work may have developed different themes or interpretations. Lastly, while saturation may have been reached with this sample to understand the complexity of DEEDs disclosure and privacy management, it is possible that additional interviews could have revealed new themes. There are numerous recommendations for future research based on the findings of this study. From a public health perspective, these findings underscore the urgent need for systemic interventions that prioritize prevention, early detection, and education rather than solely reactive treatment. Currently, many DEED interventions focus on individual treatment models, often overlooking the societal and environmental factors that contribute to DEED behaviors, disclosure, and treatment adherence [92]. Thus, future public health efforts should aim to broaden access to affordable care, implement specialized DEED training for healthcare providers, educators, and athletic coaches, and develop comprehensive educational resources for families on how to approach discussions around food and body image in an inclusive manner.

For instance, additional research should be conducted on provider (therapist)-patient communication regarding food and body image to better understand how these dynamic impacts disclosure and treatment of DEEDs, thereby tailoring interventions and improving conversations and trust in healthcare. Focus groups and/or interviews could be conducted with parents and children about their experiences with DEED, and through this, knowledge of skillful social support strategies and current coping channels.

Additionally, media literacy education is essential in mitigating the influence of social media on body image and food-related behaviors. The pervasiveness of pro-DEED content, unrealistic body standards displayed through altered and edited images, and diet culture reinforce harmful eating patterns and self-surveillance, particularly among adolescents and young adults [67]. Incorporating critical media literacy into school curricula and increasing platform accountability for harmful content can help combat these digital risk factors. Furthermore, longitudinal studies examining the long-term effects of early exposure to body image messaging and restrictive eating behaviors could provide deeper insight into how prevention efforts can be more effectively tailored.

To further broaden our understanding of DEED and its complexity, future research should also explore how different socio-economic and cultural backgrounds impact the perception and management of DEED. Given that many DEED discussions remain centered on specific demographics (e.g., white women) [93], additional studies should focus more on underrepresented populations, including racial minorities, LGBTQ + individuals, and those from diverse socio-economic backgrounds. Expanding on this, obtaining more participants, especially within these populations, and conducting a comparative analysis could be beneficial to identify differences within different communities, cities, counties, and countries.

College, a significant stressor for many young adults, is a crucial period that demands our attention and support. The prevalence of DEED is notably higher among college students, with the 12-month prevalence rates for DE ranging from 17 to 90% among female students and from 8 to 30% among male students [92]. The normalization of restrictive eating, over-exercising, and body surveillance within athletic and high-achieving environments further obscures early warning signs of DEED, allowing harmful behaviors to persist undetected [93].

Therefore, the need for more research at universities, especially within different athletic departments and with a greater number of male and non-binary students, is paramount to gather their experiences and better understand a population that is significantly impacted. Furthermore, the current study expands on this research after an unprecedented stressor, the COVID-19 pandemic, which forced individuals into lockdowns, exacerbating existing environmental, social, and familial factors that impact the development and disclosure of DE and ED and reinforced the need for more work within this area of research [94, 95]. In the context of the pandemic, college experiences can create and exacerbate psychological stressors, which are linked to DE patterns among college populations, with studies linking high levels of perceived stress and anxiety to unhealthy dietary behaviors [96, 97].

Finally, social support emerges as a significant key factor that can buffer individuals from the adverse effects of the many stressors that can lead many to DEEDs. Adolescents with strong social support networks have exhibited reduced tendencies and chances of DEEDs, while a lack of social support may contribute to the emergence of DEED behaviors. As a result, supportive relationships can help alleviate the emotional burdens caused by societal pressures related to DEEDs [98, 99]. Moreover, Bi and colleagues elaborated that individuals with strong social support networks experience reduced negative emotional impacts from media portrayals of idealized body images, increasing the risk of developing DE tendencies. However, relationships are a two-way street [100]. Many individuals who struggle with DEEDs are reluctant to seek help and withdraw from friendships due to shame, embarrassment, or a multitude of other factors. For example, Rotenberg and colleagues found that individuals suffering from BN might avoid asking for social support due to feelings of shame or embarrassment related to their eating behaviors [101]. Strong social support can alleviate these feelings, as it offers reassurance and practical aid, which enhances individuals' motivation to share their difficulties [102].

Many of these beliefs are deeply ingrained in our culture and have been exacerbated by the rise of social media and the isolation many individuals experienced during the COVID-19 pandemic. To develop interventions to support those with DEEDs and education to dismantle societal views of DEEDs as a "trend", information is needed to understand the reasons why people do not disclose their DEEDs. Specifically, what within our culture and society adds to the stigma those with DEEDs experience, and what are the things people believe should be done to help. A multitude of factors impact an individual's initiation, continuation, revelation, and remediation of DEED behaviors. Shifting from a reactive approach to a preventative public health model is essential. By addressing family dynamics, social and digital influences, and systemic barriers to care, public health efforts can foster early intervention, reduce stigma, and create healthier environments that support body acceptance and long-term recovery [103]. These findings can contribute to healthcare practitioners' understanding of DEEDs, specifically how to educate and communicate with patients about this topic. Bridging the gap between education, policy reform, and accessible healthcare is crucial in developing sustainable solutions to the widespread and often overlooked public health crisis of DEED.

## Conclusions

Privacy rules surrounding DEED are shaped by a complex set of factors, including family dynamics, social media influence, previous interactions with the healthcare system, justification factors, and the presence of comorbidities. These elements collectively inform how individuals manage, conceal, or share their experiences. Understanding why individuals experiencing DEED choose to disclose—or not disclose—their experiences, and to whom, is critical for gaining deeper insight into the personal and systemic forces that impact their support networks, coping strategies, and access to care. This knowledge can also shed light on how social and institutional structures either facilitate or hinder meaningful responses to their needs.

## Data Availability

Data sharing is not applicable to this study due to participant privacy. However, if you would like to review the material, reach out to the first author and they will be willing to talk with you more about the study and the process.

## Abbreviations

DEED: Disordered eating and eating disorders
ED: Eating disorder
DE: Disordered eating
AN: Anorexia nervosa
BN: Bulimia nervosa
ON: Orthorexia nervosa
BED: Binge eating disorder
OSFED: Other specified feeding or eating disorders
EDNOS: Eating disorder not otherwise specified
CPM: Communication privacy management
DSM-5: Diagnostic and statistical manual of mental disorders, fifth edition
OCD: Obsessive compulsive disorder
IRB: Institutional review board
BMI: Body Mass Index
MDD: Major depressive disorder
